# Profil bactériologique du pied diabétique et son impact sur le choix des antibiotiques

**DOI:** 10.11604/pamj.2015.20.148.5853

**Published:** 2015-02-17

**Authors:** Adil Zemmouri, Mohamed Tarchouli, Abdellatif Benbouha, Tarik Lamkinsi, Mustapha Bensghir, Mostafa Elouennass, Cherqui Haimeur

**Affiliations:** 1 Service de Réanimation-Anesthésie, Hôpital Militaire d'Instruction Mohammed V, Université Mohammed V, Rabat, Maroc; 2 Service de Chirurgie Viscérale I, Hôpital Militaire d'Instruction Mohammed V, Université Mohammed V, Rabat, Maroc; 3 Service de Traumatologie Orthopédie, Hôpital Militaire d'Instruction Mohammed V, Université Mohammed V, Rabat, Maroc; 4 Laboratoire de Génétique et de Biométrie, Faculté des Sciences, Université Ibn Tofail, Kénitra, Maroc; 5 Service de Bactériologie, Hôpital Militaire d'Instruction Mohammed V, Université Mohammed V, Rabat, Maroc

**Keywords:** Pied diabétique, infection, bactéries isolées, résistance bactérienne, antibiothérapie, diabetic foot, infection, isolated bacteria, bacterial resistance, antibiotherapy

## Abstract

**Introduction:**

Analyse du profil bactériologique des pieds diabétiques pris en charge à l'hôpital militaire de Rabat et son influence sur l'antibiothérapie de première intention.

**Méthodes:**

Etude prospective non randomisée étalée sur 18 mois, ayant concerné 105 patients. Après recueil des données et en attente des résultats bactériologiques nos patients ont été divisés en deux groupes: un groupe a été mis sous Amoxicilline + Acide clavulanique + Gentamycine (59 patients) et un groupe sous Ertapénème±Gentamycine (46 patients).

**Résultats:**

L’étude a regroupé 85 hommes et 20 femmes (sexe ratio = 4.26). L’âge moyen est de 64.4 ans. La gangrène a été observée chez 79% des malades; elle était humide-donc surinfectée en principe- dans 43% des cas. Par ailleurs, 67% des malades ont un chiffre de globules blancs 12000 définissant une infection sévère. L'ostéolyse a été mise en évidence chez 27% de nos patients. Parmi les différentes techniques de prélèvements: 81% ont été profonds dont 21% de biopsie osseuse per opératoire et 14% de prélèvements combinés. 42% de ces prélèvements sont poly microbiens et 21% sont stériles. Les résultats bactériologiques viennent confirmer la prédominance des bactéries aérobies à Gram positif. Le taux de remplacement de l'Ertapénème est de 22% contre un taux de 50% pour l'Amoxiclav.

**Conclusion:**

L'antibiothérapie ne doit être instaurée qu'en cas d'infection du pied diabétique diagnostiquée sur les critères cliniques établis par les consensus internationaux récents. Le respect des mesures de lutte contre la diffusion de la résistance bactérienne s'avère primordiale.

## Introduction

Le pied diabétique est le résultat de 3 mécanismes physiopathologiques intriqués: la neuropathie diabétique, l'ischémie et l'infection dont la coexistence expose à des infections sévères et étendues pouvant devenir incurables, mettant ainsi en jeu le pronostic fonctionnel voire le pronostic vital. L'infection constitue ainsi une part essentielle dans la genèse du pied diabétique, elle se caractérise la plupart du temps par la multiplicité des souches bactériennes souvent multi résistantes. L'antibiothérapie associée au traitement chirurgical demeure le pilier de la prise en charge. L'isolement des germes et le choix d'une antibiothérapie ciblée contribuent à améliorer la qualité de la prise en charge de cette maladie. Notre étude vise à analyser le profil bactériologique et son influence sur l'antibiothérapie de première intention (Amoxiclav+Genta vs Ertapénème±Genta).

## Méthodes

Notre travail est une étude prospective non randomisée réalisée sur une période de 18 mois (juillet 2012-décembre 2013) chez les patients admis pour pied diabétique et hospitalisés dans les différents services de chirurgie viscérale, traumatologie et réanimation de l'hôpital militaire d'instruction Mohammed V à Rabat. Après recueil des données épidémiologiques et cliniques; réalisation des prélèvements biologiques et explorations radiologiques; deux protocoles d'antibiothérapie de première intention ont été appliqués en attente des résultats bactériologiques. Le choix des antibiotiques s'est fait en respectant les recommandations de la société de pathologie infectieuse de langue française sur le pied diabétique (SPILF) pour la prise en charge du pied diabétique et le consensus international sur le pied diabétique[1–3], ainsi deux schémas ont été proposés pour les patients admis: Groupe 1: les patients ayant une infection plus ou moins superficielle ou une lésion chronique ont été mis sous Amoxicilline + Acide clavulanique + Gentamycine (Amoxiclav+Genta); Groupe 2: les patients ayant une lésion profonde et/ou sévère hormis ostéite ont été mis sous Ertapénème±Gentamycine (46 patients). Chez 3% de l’échantillon une conversion thérapeutique de l'amoxiclav+genta vers l'Ertapénème a eu lieu en raison d'une aggravation clinique et/ou biologique avant même d'attendre les résultats des prélèvements bactériologiques.

Dans notre contexte et en pratique, devant un membre manifestement irrécupérable vu l’état avancé de la destruction tissulaire et osseuse toute tentative de sauvetage du membre étant impossible et tout espoir de guérison sous antibiothérapie devient illusoire. Ces malades proposés d'emblée pour amputation sont mis systématiquement sous Amoxiclav+Genta en antibioprophylaxie plutôt qu'en traitement curatif et sont annexés au «groupe1» qui regroupe 59 patients au total. Les informations recueillis concernent: L’âge, le sexe, le pied concerné, les résultats du bilan biologique (glycémie à jeun, taux de leucocytes, CRP), les explorations radiologiques (radio standard, echodoppler); Les types de prélèvements, les germes isolés et leur profil de résistance (volet bactériologique); L'antibiothérapie de première intention, l'antibiothérapie instaurée, la prise en charge chirurgicale, l'oxygénothérapie hyperbare (OHB), et l’évolution. Les patients opérés ont été prélevés par différentes méthodes qui varient selon la profondeur des lésions et la présence ou non de pus. Certains patients (14%) ont bénéficié de deux prélèvements distincts combinés pour augmenter les chances d'isoler un germe et comparer les résultats. Les différèrentes méthodes de prélèvements effectués sont: Aspiration à la seringue fine (ABS): aspiration à l'aiguille d'une lésion collectée en passant par la peau saine après désinfection cutanée; Prélèvement profond per-opératoire (PPP): prélèvement simple ou biopsie tissulaire avec étude à la fois microbiologique et anatomopathologique; Biopsie osseuse per opératoire (BPO); Prélèvements superficiels (PS): écouvillonnage, prélèvement de pus superficiel; Les prélèvements superficiels et l’écouvillonnage ont été réservés exclusivement pour les patients ayant une lésion superficielle ou réalisés en combinaison avec des prélèvements profonds pour comparer les résultats. L'aspiration à la seringue a été réalisée chaque fois qu'il a eu la constatation de la présence d'une collection. La biopsie a été réalisée chaque fois qu'il s'agisse d'une extension vers l'os. Les données ont été saisies sur Microsoft Excel 2003 (Washington, USA) et l'analyse statistique a été réalisée en utilisant PASW Statistics 18.0 (Chicago, IL, USA). Les données descriptives sont présentées sous forme de médiane (IQ: Q1 - Q3) pour les variables quantitatives et sous forme d'effectifs (pourcentages) pour les variables qualitatives. Une valeur de p < 0,05 est considérée comme statistiquement significative.

## Résultats

**Figure 1:**
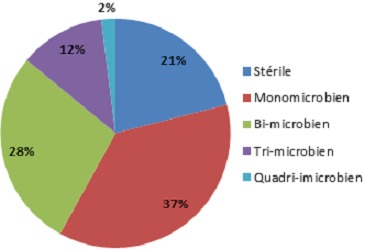
Nombre de germes

**Figure 2:**
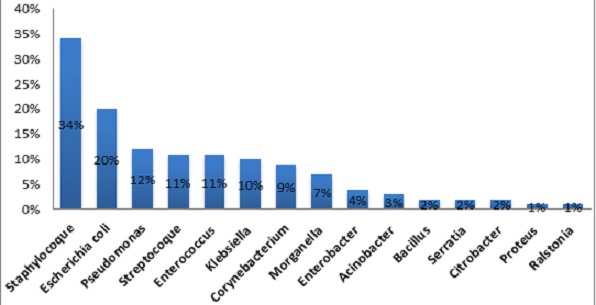
Pourcentage des bactéries isolées

Notre étude a regroupé 105 patients, 85 hommes et 20 femmes (sexe ratio = 4.26). La gangrène a été observée chez 79% de nos malades; elle était humide- (donc surinfectée en principe) dans 43% des cas. Par ailleurs, 67% ont un chiffre de globules blancs supérieur à 12000 ou inférieur à 4000 élt/mm^3^ définissant une infection sévère ou grade 4 selon le consensus international sur le pied diabétique [1, 2]. Les différentes méthodes de prélèvements bactériens réalisés sont: ABS: aspiration à la seringue fine dans 20% des cas; PPP: prélèvement profond peropératoire dans 26% des cas; BPO: biopsies osseuses peropératoires au nombre de 30 au total (21%) dont seulement 5 sont revenus stériles, c'est le «gold standard» des techniques de prélèvement [4]; PS: écouvillonnage; prélèvement de pus superficiel, le moins fiable des techniques avec recueil de la totalité de la flore commensale. Des prélèvements combinés ont été réalisés dans 14% des cas pour augmenter les chances d'isoler les germes et pour comparer les résultats des prélèvements superficiels et profonds. Le prélèvement initial à la recherche d'un germe a été réalisé dans 94% lors de la première intervention et dans 6% lors des reprises ultérieurs. Le choix de l'un ou l'autre des antibiotiques s'est fait en respectant les recommandations de la société de pathologie infectieuse de langue française sur le pied diabétique (SPILF) pour la prise en charge du pied diabétique et le consensus international sur le pied diabétique [1–3]. Parmi les différentes techniques de prélèvements: 81% ont été profonds (26% de PPP, 21% de BPO et 20% d'ABS) et 14% de prélèvements combinés. L’étude bactériologique de ces prélèvements a montré que 42% sont poly-microbiens, 37% sont mono-bactériens et 21% sont stériles (Figure 1). Les germes isolés et leur pourcentage sont représentés dans la Figure 2. Concernant le profil de résistance des germes, les staphylocoques Méticilline-sensibles représentent 72% de l'ensemble des staphylocoques isolés (34% des germes) tandis que les staphylocoques à sensibilité intermédiaire constituent 3%. Les staphylocoques Méticilline-résistants qui constituent un vrai défi thérapeutique ne représentent ainsi que 25%. L'E. Coli (20%) constamment sensible à l'Eratpénème et à l'Amikacine, semble être inconstamment sensible à la Gentamycine dans notre série (21% de résistance) tandis que l'E.Coli Amoxiclav-résistante (amoxiclav R) constitue 52% de l'ensemble des E.coli isolés. Le Pseudomonas aeruginosa (12%) vient en 3^ème^ ligne dans notre série; il est multi-sensible en général (92% des cas); donc en principe il s'agit de souches sauvages et non pas de souches hospitalières avec une résistance naturelle à l'Amoxiclav.

**Figure 3:**
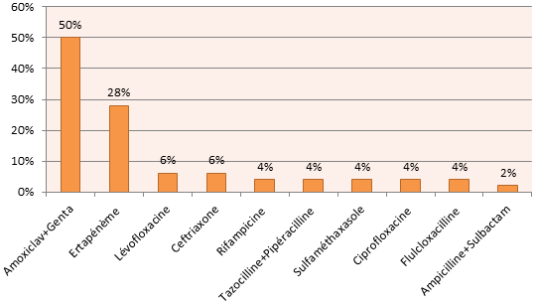
Taux de remplacement de l'amoxiclav+Genta

La résistance constatée du pyocyanique est croisée entre Imipénème, Ertapénème et Ceftazidime. Les streptocoques du groupe A constituent 58% de l'ensemble des streptocoques (11% des germes isolés). Le Streptocoque agalactiae a été isolé dans deux cas et ne pose pas de problème de résistance constatée ailleurs [5]. Le klebsiella pneumoniae (82% du genre Klebsiella) est Amoxiclav R dans 44% des cas avec une sensibilité constante à l'Ertapénème tandis que leKlebsiella oxytaca est constamment sensible à l'Amoxiclav dans notre série. Pour le reste des bactéries isolées, les Enterobacter, les Morganella morganii et les Serratia sont Amoxiclav R mais sensibles à l'Ertapénème. Les Enterocoques sont Amoxiclav sensibles mais résistants à l'Ertapénème. Alors que les Bacillus et les Proteus vulgaris sont sensibles à la fois à l'Amoxiclav et à l'Ertapénème. Les Corynebacterium species représentant 89% des corynebacteriums isolés n'ont été résistants à l'Amoxiclav que dans un cas sur huit. Enfin l'Acinetobacter baumanii isolé dans 3 situations, a été résistant à l'Ertapénème dans 2 cas. Dans 11% des cas la présence d'anaérobies a été fortement suspectée. L'Ertapénème peut être une alternative dans l'arsenal thérapeutique des infections des plaies du pied chez le diabétique notamment après une documentation microbiologique. En effet in vitro cet antibiotique est actif sur toutes les souches de Streptococcus spp, d'entérobactéries et d'anaérobies stricts et sur 89,8% des souches de Staphylocoque aureus sensibles à la Méticilline [6]. Il reste cependant actif uniquement sur 31,5% de Staphylococcus epidermidis et inactif sur Enterococcus faecalis et Pseudomonas aeruginosa. Selon Lipsky BA [7, 8] une prise unique par jour de l'Ertapénème pendant 5 jours aurait la même efficacité que l'association Piperacilline/tazobactam dans le traitement du pied diabétique infecté avec un relais par l'Amoxicalv en IV à partir du 5^ème^ jour. Selon l'indication, 41% des malades ont été mis sous Ertapénème±Genta et 56% sous Amoxiclav+Genta; 3% sous Amoxiclav+Genta puis rapidement sous Ertapénème avant d'attendre les résultats. Le taux de remplacement de l'Ertapénème est de 22% contre un taux de 50% pour l'amoxiclav (Figure 3 et 4). Concernant la prise en charge chirurgicale: 55% de nos patients ont subi une amputation en première intention, 36% une mise à plat-drainage et 9% une nécrosectomie. La reprise chirurgicale a été nécessaire dans 27% des cas. 68% de nos patients ont bénéficié de l'oxygénothérapie hyperbare comme complément thérapeutique. Le taux de décès est de 6%.

**Figure 4:**
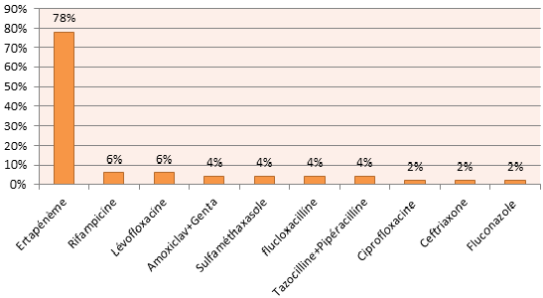
Taux de remplacement de l'Ertapénème

## Discussion

Le seul marqueur biologique reconnu par tous les auteurs par sa fiabilité dans l'infection du pied diabétique étant le nombre de globules blancs et en particulier celui des polynucléaires neutrophiles [9]. Chez 67% de nos patients on a retrouvé un nombre de globules blancs supérieur à 12000 ou inférieur à 4000 élt/mm^3^ définissant une infection sévère ou grade 4 selon le consensus international sur le pied diabétique [1–3]. Ce résultat confirme biologiquement l’état avancé de l'infection au moment du diagnostic. La protéine C reactive (CRP) est un marqueur peu fiable, faisant souvent défaut même dans les infections graves; Son évolution serait à l'inverse un bon indice pour juger la réponse au traitement anti infectieux. Le dosage a été systématiquement demandé à l'admission chez 76% de nos malades et a servi comme valeur de référence pour le suivi ultérieur de la cinétique de la CRP sous antibiothérapie. Lors des prélèvements bactériologiques notre souci permanent était d'obtenir l'isolement et l'identification des micro-organismes responsables de l'infection tout en évitant la contamination du prélèvement par la flore commensale de la peau. Plusieurs protocoles de prélèvements sont proposés par différents auteurs mais il n'existe pas de consensus quant à la meilleur technique de prélever [10, 11]. Ces techniques ont été mises au point conjointement par les cliniciens et les microbiologistes en vue d'obtenir un résultat cliniquement utile. Elles définissent la manière de prélever en fonction de la présentation clinique, le matériel à utiliser, les conditions de transport, les techniques analytiques [6, 11]. Dans notre étude quatre méthodes de prélèvements ont été appliqués; le prélèvement superficiel et l’écouvillonnage ont été strictement réservés aux plaies superficielles bien préparées; la grande majorité de nos prélèvements ont été profonds (81%) et le choix d'une technique de prélèvement profond a été décidé en per opératoire par le chirurgien en fonction de son constat des dégâts; l'aspiration à la seringue fine chaque fois qu'on a constaté la présence d'une collection, la biopsie osseuse (30 au total dont 5 revenues stériles) a été réalisée lorsqu'il s'agit d'une extension osseuse, le prélèvement tissulaire profond (biopsie tissulaire) pour le reste. Pour augmenter les chances d'isoler un germe et pour comparer les résultats on a opté dans notre étude pour combiner les prélèvements dans certaines situations (14% cas). Les hémocultures n'ont concerné que les patients ayant séjourné en réanimation et sont revenues toutes stériles. Le prélèvement bactériologique a été réalisé d'emblée chez 94% de nos patients opérés. Pour le reste il a été effectué lors de la reprise chirurgicale en raison d'une mauvaise évolution.

Les exigences particulières des cultures en anaérobiose nous ont conduits à consacrer notre étude à l'isolement des germes aérobies stricts alors que l'association des deux est souvent le cas [4, 12]. Bien que les anaérobies stricts soient largement inculpés dans la surinfection du pied diabétique surtout ischémié, ils demeurent néanmoins sensibles aux antibiotiques utilisés classiquement dans cette pathologie et donc en pratique leur isolement est cliniquement peu utile [13]. Dans 11% de nos prélèvements leur présence a été fortement suspectée sur uniquement l'examen direct. La surinfection du pied diabétique est en principe multi bactérienne [14]. Dans notre étude 42% de nos prélèvement ont été poly microbiens (2, 3, voir 4 bactéries) (Figure 1); les prélèvements stériles constituent 21%. Concernant les résultats des prélèvements, notre étude vient confirmer la prédominance des bactéries à gram positive [15, 16] qui reste cependant non universelle [4]. Les staphylocoques viennent en tête dans toutes les études réalisées sur ce sujet avec des taux variables. Nous avons pu l'isoler dans 34% des cas, il s'agit essentiellement de Staphyloccus aureus dont 91% sont Méticilline sensibles (SAMS). Les Staphylocoques Méticilline résistants (SARM) constituent actuellement un problème de première importance [17]. Leur isolement n'est pas obligatoirement synonyme de virulence accrue [12]. E.Coli vient en 2^ème^ position (20%); si elle est constamment sensible à l'Ertapenème, on a été surpris par l’émergence de souches résistantes à l'Amoxiclav (52%) et plus ou moins à la Gentamycine, ces dernières restent en revanche constamment sensibles à l'Amikacine. Le Pseudomonas aeruginosa isolé dans 12% des cas a un rôle pathogène discutable [7]. Il s'agit dans 92% des cas de souches multi-sensibles donc extrahospitalières en principe. Les souches de pyocyaniques multi-résistantes notamment à l'Imipenème et aux céphalosporines de 3^ème^ génération (y compris la Ceftazidime) sont isolées volontiers lors des reprises chirurgicales et lors des hospitalisations de longue durée. L’émergence des pyocyaniques dans les prélèvements serait en rapport avec des erreurs de manipulations et/ou une contamination [18]. Les Streptocoques et les entérocoques occupent la 4^ème^ position avec 11% pour chacun d'eux. Les streptocoques du groupe A prédominent (58%) alors que les streptocoques β hémolytiques du groupe B et en particulier le Streptocoque agalactiae est moins isolé. Ce dernier se caractérise par sa sensibilité aux antibiotiques classiquement prescrits dans cette pathologie et ne pose pas encore de problème de résistance rencontrée ailleurs [5]. En dehors de la pénicilline G nos streptocoques et nos entérocoques sont multi-sensibles aux antibiotiques testés (Enterococcus possède une résistance naturelle aux carbapénèmes). Les kelbsielles (10%)-dont kelbsiella pnemoniae constitue 82%)-sont constamment sensible aux carbapénèmes, en revanche la sensibilité constante de Klebsiella oxytoca à l'Amixclav contraste avec une résistance accrue de Klebsiellapneumoniae (44% de résistance). Morganella (7%), Enterobacter (4%), naturellement résistantes à l'Amoxiclav demeurent toutes sensibles à l'Ertapénème. Pour les autres enterobactéries: Serratia (2%) et bacillus (2%) demeurent sensibles à la fois à l'Amoxiclav et à l'Ertapénème alors que le Corynebacterium species (89% des corybacteriums) est Amoxiclav résistante. L'Acinetbacter baumanii isolé à 3 reprises a été résistant à l'Ertapénème.

Il est conseillé de ne pas prendre en compte en première intention des germes les moins virulents ou des commensaux (Staphylocoques coagulase négative, corynebactéries, Pseudomonas aeruginosa,enterocoques) [7, 15]. Ceux-ci peuvent se révéler cependant comme des pathogènes opportunistes [19]. En cas de doute, les prélèvements doivent être répétés et ces bactéries seront prises en considération si elles sont isolées à plusieurs reprises ou si l’état septique du patient est inquiétant. L'incidence relativement faible des bactéries multi-résistantes notamment des SAMR dans notre série par rapport à la plupart des autres séries publiées serait vraisemblablement due en grande parti à la stratégie chirurgicale privilégiée par nos équipes d'emblée [20–22]. Cette attitude d'intervention rapide et agressive ne laissant pas assez de temps à une éventuelle colonisation par la flore nosocomiale pourvoyeuse par la suite d'une éventuelle infection à bactéries multi résistantes; d'autant plus le faible recours dans notre contexte local à une antibiothérapie intempestive sélectionnant les souches résistantes avant l'admission à l'hôpital. L'antibiothérapie de première intention a été rectifiée en fonction non seulement des résultats de l'antibiogramme mais aussi en fonction de l’état local du membre, de l’état général du patient, des découvertes per opératoires, en particulier une éventuelle extension osseuse, et de l’évolution. En cas d'ostéite les antibiotiques ayant une bonne pénétration osseuse [23, 24] ont été prescrits si un traitement à visée curative a été décidé. Pour des simples raisons de disponibilité, les antibiotiques privilégiés chez nous dans ce contexte ont étéla Rifampicine et la Lévofloxacine, et en second lieu la Ciprofloxacine, le Cotrimoxazole (Figure 3 et 4). Pour la Vancomycine largement utilisée chez nous chez les sujets immunocompétents dans les ostéites et les ostéo-arthrites septiques chroniques à staph méti R, elle n'a pas eu sa place dans l'arsenal thérapeutique de l'ostéite à staphylococcus aureus meti R en raison du nombre isolé réduit de ce germe chez nous ( 3 cas) chez des malades amputés d'emblée. Par ailleurs 70% de nos patients ont subi un acte chirurgical dans les 24h suivant l'admission. L'amputation en première intention a concerné 55% de nos patients tandis que la nécrosectomie-débridement n'a concerné que seulement 9%. Le reste a subit un drainage-mis à plat en raison de la présence d'une collection. Le taux excessif d'amputation devance celui observe dans les pays occidentaux [25–27]. Le pourcentage “tragique” de l'amputation d'emblée chez nous s'explique par l’évolution avancée des lésions trophiques au moment de l'admission interdisant toute tentative de sauvetage du membre. Concernant l’évolution post opératoire immédiate, elle a été jugée insuffisante voire inquiétante chez 33% de nos patients dont 25% ont subi une reprise chirurgicale dans un délai moyen de 10 jours. Le taux faible de décès 6/105 (5,7%) vient prouver le pronostic potentiellement fonctionnel de cette pathologie. Il est plutôt proche de celui rapporté par des auteurs occidentaux [28, 29].

## Conclusion

L'incidence du pied diabétique ne cesse d'augmenter constituant ainsi un défi croissant pour la santé publique. La résistance des germes isolés aux antibiotiques proposés en première intention pose un sérieux défit thérapeutique. Devant une écologie bactérienne polymorphe et devant la résistance constatée aux antibiotiques, la collaboration entre anesthésiste réanimateur, chirurgien et biologiste est primordiale pour une meilleure prise en charge du pied diabétique.

## References

[1] Apelqvist J, Bakker K, Van Houtum WH, Schaper NC (2008). Practical guidelines on the management and prevention of the diabetic foot: based upon the International Consensus on the Diabetic Foot (2007) Prepared by the International Working Group on the Diabetic Foot. Diabetes Metab Res Rev..

[2] Schaper NC, Apelqvist J, Bakker K (2003). The international consensus and practical guidelines on the management and prevention of the diabetic foot. CurrDiab Rep..

[3] Société de Pathologie Infectieuse de Langue Française (2007). Prise en charge du pied diabétique infecté. Med Mal Infect..

[4] Senneville E (2008). Infection et pied diabétique. Rev Med Interne..

[5] Loan CA, Legout L, Assal M, Rohner P (2005). Infections sévères à Streptococcus agalactiae du pied diabétique: Rôle délétère du Streptococcus agalactiae?. Presse Med..

[6] Sotto A, Lemaire X, Jourdan N, Bouziges N (2008). Activité in vitro de l'értapénème vis-à-vis de souches bactériennes isolées de plaies infectées du pied chez des patients diabétiques. Med Mal Infect..

[7] Lipsky BA, Armstrong DG, Citron DM, Tice AD (2005). Ertapenem versus piperacillin/tazobactam for diabetic foot infections (SIDESTEP): prospective, randomised, controlled, double-blinded, multicentre trial. Lancet..

[8] Lipsky BA, Giordano P, Choudhri S, Song J (2007). Treating diabetic foot infections with sequential intravenous to oral moxifloxacin compared with piperacillin-tazobactam/amoxicillin-clavulanate. J Antimicrob Chemother..

[9] Armstrong DG, Lavery LA, Sariaya M, Ashry H (1996). Leukocytosis is a poor indicator of acute osteomyelitis of the foot in diabetes mellitus. J Foot Ankle Surg..

[10] Lipsky BA, Berendt AR, Cornia PB, Pile JC (2012). 2012 Infectious Diseases Society of America clinical practice guideline for the diagnosis and treatment of diabetic foot infections. Clin Infect Dis..

[11] O'Meara S, Nelson EA, Golder S, Dalton JE (2006). Systematic review of methods to diagnose infection in foot ulcers in diabetes. Diabet Med..

[12] Johnson S, Lebahn F, Peterson LR, Gerding DN (1995). Use of an anaerobic collection and transport swab device to recover anaerobic bacteria from infected foot ulcers in diabetics. Clin Infect Dis..

[13] Gerding DN (1995). Foot infections in diabetic patients: the role of anaerobes. Clin Infect Dis..

[14] Hunt JA (1992). Foot infections in diabetes are rarely due to a single microorganism. Diabet Med..

[15] Lipsky BA, Berendt AR, Deery HG, Embil JM (2004). Diagnosis and treatment of diabetic foot infections. Clin Infect Dis..

[16] Lipsky BA, Sheehan P, Armstrong DG, Tice AD (2007). Clinical predictors of treatment failure for diabetic foot infections: data from a prospective trial. Int Wound J..

[17] Tentolouris N, Jude EB, Smirnof I, Knowles EA (1999). Methicillin-resistant Staphylococcus aureus: an increasing problem in a diabetic foot clinic. Diabet Med..

[18] Hartemann-Heurtier A, Robert J, Jacqueminet S, Ha Van G (2004). Diabetic foot ulcer and multidrug-resistant organisms: risk factors and impact. Diabet Med..

[19] Bessman AN, Geiger PJ, Canawati H (1992). Prevalence of Corynebacteria in diabetic foot infections. Diabetes care..

[20] Couret G, Desbiez F, Thieblot P, Tauveron I (2007). Emergence des infections monomicrobiennes à staphylocoque doré méticilline-résistant dans les ostéites du pied diabétique (étude rétrospective de 48 cas). Presse Med..

[21] Kandemir O, Akbay E, Sahin E, Milcan A (2007). Risk factors for infection of the diabetic foot with multi-antibiotic resistant microorganisms. J Infect..

[22] Lipsky BA (2007). Diabetic foot infections: microbiology made modern?. Array of hope. Diabetes care..

[23] Edmonds M, Foster A (2004). The use of antibiotics in the diabetic foot. Am J Surg..

[24] Lipsky BA, Berendt AR (2000). Principles and practice of antibiotic therapy of diabetic foot infections. Diabetes Metab Res Rev..

[25] Shojaiefard A, Khorgami Z, Mohajeri-Tehrani MR, Larijani B (2013). Large and deep diabetic heel ulcers need not lead to amputation. Foot Ankle Int..

[26] Van Battum P, Schaper N, Prompers L, Apelqvist J (2011). Differences in minor amputation rate in diabetic foot disease throughout Europe are in part explained by differences in disease severity at presentation. Diabet Med..

[27] Weledji EP, Fokam P (2014). Treatment of the diabetic foot - to amputate or not?. BMC Surg..

[28] Rayman G, Krishnan ST, Baker NR, Wareham AM (2004). Are we underestimating diabetes-related lower-extremity amputation rates? Results and benefits of the first prospective study. Diabetes care..

[29] Richard JL, Lavigne JP, Got I, Hartemann A (2011). Management of patients hospitalized for diabetic foot infection: results of the French OPIDIA study. Diabetes Metab..

